# Taurine Alleviates Cadmium-Induced Hepatotoxicity by Regulating Autophagy Flux

**DOI:** 10.3390/ijms24021205

**Published:** 2023-01-07

**Authors:** Yuntian Duan, Yumeng Zhao, Tao Wang, Jian Sun, Waseem Ali, Yonggang Ma, Yan Yuan, Jianhong Gu, Jianchun Bian, Zongping Liu, Hui Zou

**Affiliations:** 1 College of Veterinary Medicine, Yangzhou University, Yangzhou 225009, China; 2 Joint International Research Laboratory of Agriculture and Agri-Product Safety of the Ministry of Education of China, Yangzhou 225009, China; 3 Jiangsu Co-Innovation Center for Prevention and Control of Important Animal Infectious Diseases and Zoonoses, Yangzhou 225009, China

**Keywords:** cadmium, taurine, hepatocytes, autophagy flux, SNARE

## Abstract

Our previous studies have confirmed that cadmium (Cd) exposure causes hepatotoxicity; it also induces autophagy and blocks the autophagy flux. Therefore, we hypothesized that Cd hepatotoxicity could be alleviated through nutritional intervention. Taurine (Tau) has various biological functions such as acting as an antioxidant, acting as an anti-inflammatory, and stabilizing cell membranes. In order to explore the protective effect and internal mechanism of Tau on Cd-induced hepatotoxicity, normal rat liver cell line BRL3A cells were treated with Cd alone or in combination with Tau to detect cell injury and autophagy-related indexes in this study. We found that Tau can alleviate Cd-induced cell-proliferation decline and morphological changes in the cell. In addition, Tau activates autophagy and alleviates the blockage of Cd-induced autophagy flux. In this process, lysosome acidification and degradation were enhanced, and autophagosomes were further fused with lysosomes. Then, we found that Tau alleviated autophagic flux block by promoting the transfer of membrane fusion proteins STX17 and SNAP29 to autophagosomes and the translocation of VAMP8 to lysosomes, which in turn attenuated the hepatocyte injury induced by Cd exposure. This will further reveal the hepatotoxicity mechanism of Cd and provide the theoretical basis for the prevention and treatment of Cd poisoning.

## 1. Introduction

Cadmium (Cd) is a poisonous heavy metal. The level of Cd in the environment is rising due to industrialization and urbanization, which have a negative effect on ecosystems, human health, and food safety [1]. More than one-third of the body’s total Cd reserves are found in the liver, which is also extremely vulnerable to Cd-induced cytotoxicity [2]. Numerous investigations have shown that Cd exposure raises the level of alanine aminotransferase (ALT) and aspartate aminotransferase (AST), two crucial biological markers of liver impairment [3]. Oxidative stress, inflammation, mitochondrial damage, and apoptosis have been the main topics of recent studies on the mechanism of action of Cd-induced liver injury. Our previous studies have found that the destruction of autophagy is also an important toxic mechanism of Cd [4,5]. These reports on the mechanisms of Cd toxicity have laid the foundation and pointed the way for research to deal with Cd poisoning. Autophagy is a significant biological process that eukaryotic cells use to maintain homeostasis in living organisms. Autophagy is a dynamic rather than a static process, with various steps of autophagy occurring at the same time, whereas autophagic flux refers to the movement of the material to be degraded throughout the autophagic system [6]. When any step of autophagy goes wrong, it can cause autophagic flux damage. A growing body of research has discovered that Cd affects autophagy, primarily in the form of intermediate concentrations that trigger cellular autophagy and apoptosis, whereas high dosages (50 μM) of Cd cause cell necrosis [7]. Although there is ongoing discussion over the role of autophagy in Cd damage, most research has demonstrated that activated autophagy mostly protects against the toxic damage caused by Cd. It has also been found that Cd causes DNA damage and elevated intracytoplasmic calcium ion concentration in endothelial cells, which initiates autophagy, endoplasmic reticulum stress, and mitochondrial membrane depolarization while activating multiple cell death signals [8]. Additionally, membrane trafficking, the development of autophagic precursor structures, the extension of phagocytic vesicles, and autophagosome–lysosome fusion are essential for the occurrence of autophagy; these processes are tightly controlled by membrane-associated proteins [9]. Current studies have confirmed that soluble NSF attachment protein receptor (SNARE) proteins play an important regulatory role in both autophagosome formation and autophagosome–lysosome fusion. The formation of a quadruple helix SNARE complex by STX17, SNAP29, and VAMP8, which facilitates autophagosome–lysosome fusion, is one of the most significant processes. However, VAMP8 is localized to the lysosome, and STX17 and SNAP29 are found in the autophagosome. Previous research has demonstrated that one of the most effective ways to treat lead or Cd poisoning is by activating autophagy and restoring autophagic flux [10,11,12,13]. The connection between autophagy and apoptosis, inflammation, and other physiological phenomena is gradually being revealed as more molecular mechanisms regulating autophagy are found, but more research is required to understand the targeting and regulation mechanisms of autophagy and its effects on the organism.

Taurine (Tau) is an essential amino acid for human health; its functions include maintaining healthy intracellular and extracellular osmotic pressure balance, stabilizing cell membranes, regulating cellular calcium homeostasis, and acting as an anti-inflammatory and antioxidant [14,15,16]. The current research on the protective effects of Tau on heavy metal poisoning is very extensive. Numerous experiments have proven that Tau has clear alleviating effects on the damage caused by heavy metals such as cadmium, manganese, and lead. The main mechanisms of action are alleviating oxidative damage, reducing apoptosis, and alleviating the dysfunction of target organs. According to Hwang et al. [17], Tau can not only significantly decrease the amount of Glutathione (GSH) and Malondialdehyde (MDA) in the liver, but it can also increase the amount of Cd in the feces of Cd-stained rats, preventing accumulation in the body.

Additionally, in vitro studies have shown that Tau reduces the oxidative harm caused by Cd chloride to rat hepatocytes. Tau supplementation reduces aberrant morphological alterations in renal glomeruli and proximal tubules in a mouse model of arsenic poisoning [18]. Tau is crucial for ion transport, maintaining excitatory conduction in neurons, and protecting the brain from oxidative damage brought on by aluminum [19]. The significance of Tau in reducing heavy metal toxicity has been studied in this research project. However, the intrinsic mechanism by which Tau reduces Cd hepatotoxicity via regulating autophagy has not been thoroughly understood. Therefore, utilizing the rat BRL3A cell line as a model, this study aimed to explore further the precise mechanism by which Tau reduces Cd-induced hepatocyte injury by regulating autophagy, specifically the fusion and degradation phases.

## 2. Results

### 2.1. Tau Can Alleviate Cd-induced BRL3A Cell Damage

**Figure 1:**
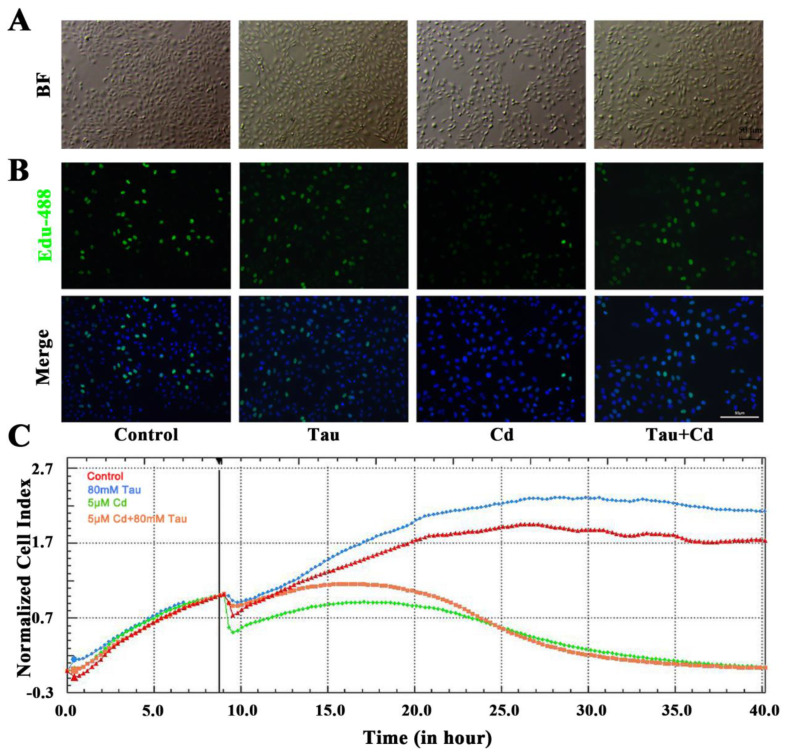
Tau can alleviate Cd-induced BRL3A cell damage. (**A**) Cd and Tau on cell morphology in BRL3A cells (scale bar = 50 μm). BRL3A cells were treated with or without 80 mM Tau or 5 μM Cd for 6 h; then, they were viewed and photographed under a Leica DMIRB. (**B**) Effect of Tau and Cd on cell proliferation in BRL3A cells (scale bar = 50 μm). (**C**) Effect of Cd and Tau on cell index in BRL3A cells.

In total, 5 μM Cd and 80 mM Tau were applied to BRL3A cells separately or together for 6 h. Microscopically, morphological variations in cells were seen. The findings demonstrated that the cell morphology dramatically changed after 6 h of Cd treatment, with bigger gaps and cell crinkling. Limited cell morphology variations were observed in the Tau and Cd co-treated group than in the Cd-alone treatment group (Figure 1A). Proliferation of BRL3A cells was detected by the Edu-488 assay, and the proliferating cells showed green fluorescence under fluorescence microscopy (Figure 1B). The number of Edu-positive cells was significantly reduced in the Cd group compared with the Control, indicating that Cd inhibits cell proliferation. The number of edu-positive cells was increased in the Tau and Cd co-treatment group compared with the Cd group. Finally, cell index changes in BRL3A cells ware observed. Cell index increased in the Tau-alone group and dramatically slowed down in the Cd-alone group compared to the Control, according to the results. Cell index also increased in the Tau and Cd co-treated group compared to the Cd-alone group (Figure 1C). These findings imply that Tau can effectively alleviate Cd-induced damage to BRL3A cells.

### 2.2. Tau Can Alleviate Cd-induced Autophagy Flux Arrest in BRL3A Cells

**Figure 2:**
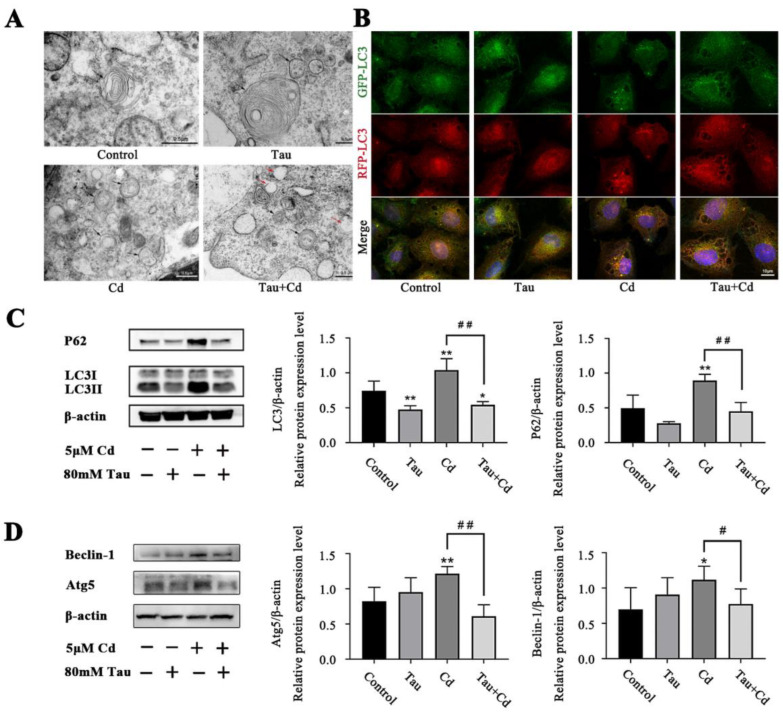
Tau can alleviate Cd-induced autophagy flux arrest in BRL3A cells. (**A**) Effect of Tau and Cd on autophagosomes and autolysosomes in BRL3A cells (scale bar = 0.5 μm). The autophagosomes and autolysosomes were marked with black and red arrows. (**B**) Effect of Tau and Cd on RFP-GFP-LC3 fluorescent protein expression in BRL3A cells (scale bar = 10 μm). (**C**) Effect of Tau and Cd on the levels of LC3 and P62 proteins in BRL 3A cells. (**D**) Effect of Tau and Cd on the protein levels of Atg5 and Beclin-1 in BRL 3A cells. Compared with the Control group, * *p* < 0.05 and ** *p* < 0.01. Compared with the Cd-treated group, # *p* < 0.05 and ## *p* < 0.01.

BRL3A cells were treated with 5 μM Cd and 80 mM Tau either separately or together during 6 h. Transmission electron microscopy was used to detect the alterations in autophagic vesicles. The findings demonstrated that autophagic vesicles, which had a bilayer membrane structure and wrapped the material to be degraded, increased in the cells of the Cd-alone treatment group compared to the Control group; they decreased in the Tau and Cd co-treatment group compared to the Cd-alone treatment group, whereas the number of autolysosomes increased (Figure 2A). RFP-GFP-LC3 tandem fluorescent protein labeling of cells is a common method to detect autophagic flux, and the results are shown in Figure 2B. There were few fluorescent aggregates and consistent expression of green fluorescence and red fluorescence in the Control group cells, indicating a low level of autophagy and less autophagosome formation. Compared with the Control, there was an increase in yellow fluorescent aggregates in the Cd-treated group cells, indicating a significant increase in autophagic water and autophagic flux block. However, the yellow fluorescent clusters were significantly reduced after co-treatment with Tau, indicating that the Cd-induced autophagic flux block in BRL3A cells was alleviated. Finally, Western blot analysis was used to assess protein expression levels for LC3, P62, Atg5, and Beclin-1. According to the results, the relative expression of LC3II was highly significantly reduced in the Tau-alone group compared to the Control (*p* < 0.01), whereas the relative expression of Atg5, LC3II, P62, and Beclin-1 was highly significantly increased in the Cd-alone group (*p* < 0.01, *p* < 0.05). In contrast to the Cd-alone group, the relative expressions of Atg5 and LC3 decreased significantly in Tau and Cd combined treatment group (Figure 2C,D). These findings imply that Tau can effectively alleviate the autophagic flux obstruction caused by Cd.

### 2.3. Tau Can Facilitate the Union of the Lysosome and the Autophagosome and Improve the Lysosome’s Capacity for Acidification and Degradation

**Figure 3:**
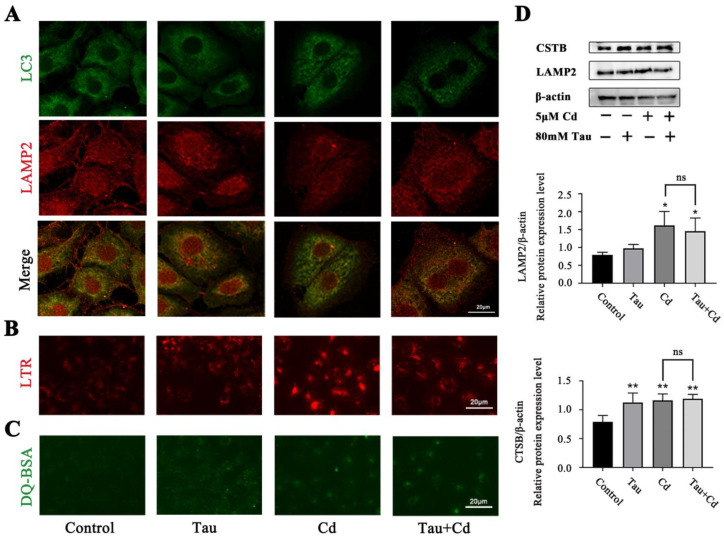
Tau can facilitate the union of the lysosome and the autophagosome and improve the lysosome’s capacity for acidification and degradation. (**A**) Effect of Tau and Cd on the co-localization of LC3 with LAMP2 in BRL3A cells (scale bar = 20 μm). (**B**) Effect of Tau and Cd on lysosomal acidity in BRL3A cells (scale bar = 20 μm). (**C**) Effect of Tau and Cd on lysosomal acidity in BRL3A cells (scale bar = 20 μm). (**D**) Effect of Tau and Cd on the protein levels of LAMP2, CTSB in BRL 3A cells. Cells were treated with 80 mM Tau and/or 5 μM Cd for 6 h; then, the proteins levels of Atg5 and Beclin-1 were analyzed by Western blotting. Compared with the Control group, * *p* < 0.05 and ** *p* < 0.01. Compared with the Cd-treated group, ns > 0.05.

BRL3A cells were treated with 80 mM Tau and 5 μM Cd alone or in combination for 6 h. The co-localization of LC3 with LAMP2 was detected by immunofluorescence. The results showed that the co-localization of LC3 with LAMP2 was reduced in the Cd-alone group compared with the Control; the co-localization of the Tau with Cd co-treated group was increased compared with the Cd-alone group (Figure 3A), indicating that Tau alleviated the Cd-induced blockage of autophagosome–lysosome fusion in BRL3A cells. Next, this experiment used LTR fluorescence staining to detect the acidic environment of lysosomes. The results are shown in Figure 3B. Compared with the Control group, the fluorescence intensity of the Cd-alone treatment group was significantly enhanced, and the lysosomes were aggregated, indicating the acidification of the lysosomes. The fluorescence intensity of the Tau-alone treatment group was also enhanced, indicating that Tau caused the acidification of the lysosomes. Compared with the Cd-alone treatment group, the fluorescence intensity of the Tau and Cd co-treatment group was weakened, and the lysosomes were still dispersed to the around the nucleus. To investigate the effect of Tau and Cd on the lysosomal degradation function of BRL3A cells, the cells were first incubated in DQ-BSA-Green working solution for 2 h and then treated with 80 mM Tau and 5 μM Cd alone or in combination for 6 h. The cells were photographed under a fluorescence microscope. The results showed that the fluorescence fragment release was increased in the Tau-alone group compared with the Control group, indicating enhanced degradation of lysosomes. The fluorescence intensity was also significantly enhanced in the Cd-alone group due to acidification of lysosomes; the fluorescence fragment release was increased in the Tau and Cd co-treated group compared with the Cd-alone group (Figure 3C). Western blot was performed to detect the expression levels of CTSB and LAMP2. The results showed that the relative expression of LAMP2 was significantly higher in the Cd-alone group compared to the Control (*p* < 0.05), and the relative expression of CTSB was highly significantly higher in both the Tau- and Cd-alone groups (*p* < 0.01). Compared with the Cd-alone group, the relative expression of CTSB and LAMP2 in both the Tau and Cd co-treated groups did not change significantly (Figure 3D). According to the aforementioned findings, Tau may encourage the fusion of lysosomes and autophagosomes and may improve the acidification and degradation capabilities of lysosomes.

### 2.4. Tau Promotes the Transfer of STX17 and SNAP29 to Autophagosomes and VAMP8 to Lysosomes

**Figure 4:**
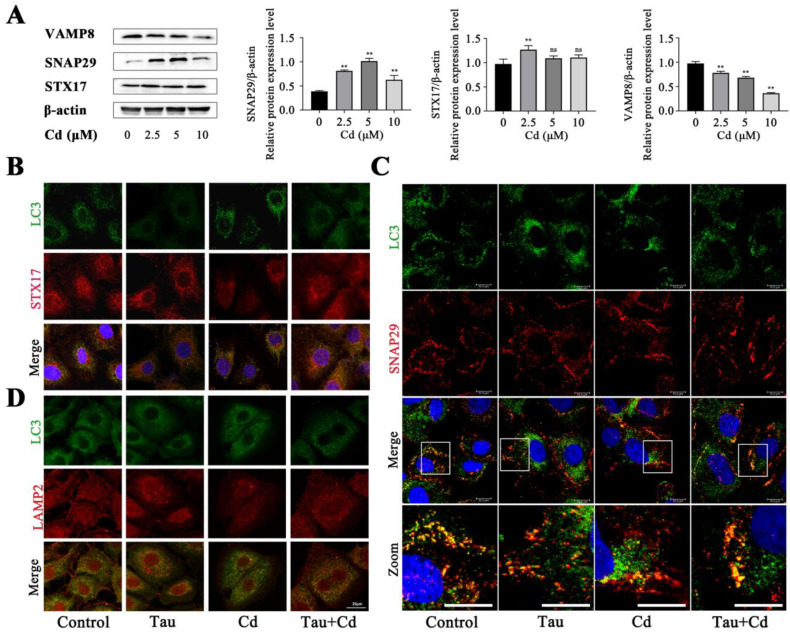
Tau promotes the transfer of STX17 and SNAP29 to autophagosomes and VAMP8 to lysosomes. (**A**) Effects of Cd on the levels of membrane-fusion-associated proteins in BRL3A cells. Cells were treated with varying concentrations of Cd (0, 2.5, 5, and 10 μM) for 6 h, then the proteins levels of STX17, SNAP29, VAMP8 were analyzed by Western blotting. Compared with the Control group, ns > 0.05, ** *p* < 0.01. (**B**,**C**) Effect of Tau and Cd on the co-localization of LC3 with STX17 and SNAP29 in BRL3A cells (scale bar = 20 μm). (**D**) Effect of Tau and Cd on the co-localization of VAMP8 with LAMP2 in BRL3A cells (scale bar = 20 μm).

To detect Cd’s effect on membrane fusion-related proteins’ expression, BRL3A cells were treated with different concentrations of Cd (0, 2.5, 5, and 10 μM) for 6 h. The expression levels of membrane fusion-related proteins STX17, SNAP29, and VAMP8 were detected by Western blot. The results are shown in Figure 4A. Compared with the blank Control group, there was no significant change in the overall expression of STX17, and the relative expression was only highly significantly increased at 2.5 μM Cd treatment for 6 h (*p* < 0.01). The relative expression of SNAP29 was increased overall (*p* < 0.01). The relative expression of VAMP8 was decreased in a concentration-dependent manner (*p* < 0.01). To further investigate the molecular mechanism of Cd-induced autophagic flux blockage in BRL3A cells from the perspective of membrane fusion and the mechanism of Tau alleviating Cd-induced autophagic flux damage in BRL3A cells, the co-localization of LC3 with STX17 was detected by immunofluorescence after treatment. The results showed that the co-localization of LC3 with STX17 was reduced in the Cd-alone group compared with the Control; co-localization was increased in the Tau and Cd co-treatment group compared to the Cd-alone group (Figure 4B). Next, after treatment, LC3 co-localization with SNAP29 was detected by immunofluorescence assay. The results showed that the co-localization of LC3 with SNAP29 was reduced in the Cd-alone treatment group compared with the Control. The co-localization of Tau with the Cd co-treatment group was increased compared with the Cd-alone treatment group (Figure 4C). Finally, the lysosomal localization of VAMP8 was investigated by detecting the co-localization of VAMP8 with LAMP2 by immunofluorescence after treatment. The results showed that the co-localization of VAMP8 with LAMP2 was reduced in the Cd-alone treatment group compared with the Control. Compared with the Cd group, the co-localization of the Tau and Cd group increased (Figure 4D). The above results indicated that Tau could promote the transfer of membrane-fusion proteins STX17 and SNAP29 to autophagosomes and VAMP8 to lysosomes.

## 3. Discussion

Cd contamination of soil and food is very common, and there have been numerous reports of Cd contamination incidents [20,21]. Cd damages the liver parenchyma and impairs liver function [22]. It also alters hepatic lipid metabolism and causes lipid droplet deposition, which promotes conditions such as nonalcoholic steatohepatitis [23]. At present, it is considered that the main mechanisms of Cd-induced liver damage are as follows: it causes oxidative stress, resulting in an increased peroxide level, which in turn leads to inflammation, endoplasmic reticulum stress, and other further damage [24]. It induces apoptosis through various pathways such as FAS/FASL and endoplasmic reticulum [25]. Tau, an antioxidant naturally present in all living things, can stabilize cell membranes and maintain osmotic pressure homeostasis, among other things. Tau also has important effect in reducing the harm caused by heavy metals in the animal body. According to Prasenjit Manna et al. [26], Tau enhanced the antioxidant capacity of kidney tissues and had a mitigating effect on Cd-induced nephrotoxicity. Tau also improved the cardiac dysfunction brought on by Cd in mice and the quantity and viability of sperm in the testicles [27,28]. AML12 cells of the mouse hepatocyte line were damaged by 5 μM Cd exposure [29]. The current experiment’s findings are in line with those of the previous study, showing that Tau mitigated the morphological changes and inhibited cell proliferation brought on by Cd. It suggests that Tau can be further researched as a viable medicine for Cd poisoning prevention and therapy.

Autophagy is a dynamic and complex process; impairment of any of its elements can impact autophagic flux. Cd elevates the level of autophagy in rat BRL3A cells [30] and results in higher levels of autophagy and blocked autophagic flux in mouse hepatocytes AML12 cells [29]. Ni Gao et al. [31] found that Tau can inhibit hepatic autophagy by activating the PPARγ-mTORC2 signaling pathway. When calcium oxalate crystals damage renal tubular epithelial cells, Tau can activate the Akt/mTOR signaling pathway to block ROS-dependent autophagy [32]. Tau can also reduce the production of the LC3-II protein and the fluorescence of GFP-LC3 sites in PK-15 cells, which attenuates the effects of Hercotoxin A-induced autophagy [33]. Tau has also been found to promote autophagy; for example, increased TFEB nuclear translocation induced autophagic clearance in Tau-treated mouse adipocytes [34]. Tau activates autophagy by inhibiting mTOR signaling and thus decreasing phosphorylation of ULK1 and ATG13 [35]. The microtubule-associated protein LC3 is an autophagosome ortholog of yeast Atg8, which is associated with autophagosome membranes after processing, and is modified via a ubiquitination-like system. The LC3 is now widely used to monitor autophagy, which is a good early marker for the formation of autophagosomes. Thus, the increased expression of LC3-II is associated with autophagy induction. In the present study, in the Tau-alone treatment group, LC3 expression decreased significantly, indicating a decrease in the number of autophagosomes, which was consistent with the view that Tau could inhibit autophagy in previous studies. Compared with the Cd group, the Tau and Cd co-treatment group showed a decrease in the number of autophagosomes, an increase in the number of autophagic lysosomes, a significant decrease in the expression of autophagy-related proteins LC3, P62, Atg5, and Beclin-1, and a decrease in RFP-GFP-LC3 yellow puncta, indicating that Tau can alleviate Cd-induced autophagic flux damage and also inhibit pre-autophagy and, thus, reduce autophagosome production.

As the most important organelle in the autophagic process, the normal morphology and function of lysosomes have an impact on autophagy [36]. When cells are subjected to external environmental stimuli or starvation conditions, mTORC1 detaches from lysosomes and induces autophagy, whereas TFEB dephosphorylates and migrates to the nucleus, activating the lysosomal biogenesis and autophagy regulatory mechanisms [37]. The findings of this study suggested that Tau could speed up the autophagic process and encourage the fusion of autophagosomes with lysosomes, because the expression of LC3 and P62 was reduced and the co-localization of LC3 and LAMP2 was increased in the Tau and Cd co-treatment group compared to the Cd-alone group. In the Tau and Cd co-treatment group, the lysosome was less acidic than that in the Cd-alone treatment group, and the aggregation of lysosomes was alleviated. This is not only related to Tau-inhibiting pre-autophagy but also possible because Tau promotes the fusion of autophagosomes and lysosomes. The lysosomes transported to the nucleus were fully utilized by fusion with autophagosomes, and then some hydrolases were consumed. This may also account for the rise in DQ-BSA-Green fluorescence fragmentation, primarily due to an increase in the number of lysosomes that break down autophagosomes. After the addition of Tau, the changes of LAMP2 and CTSB were not significant, suggesting that Tau did not promote the autophagy process mainly by regulating the lysosomes, and there might be other action targets.

SNARE proteins mediate autophagy-related membrane fusion mechanisms. Some SNAREs perform particular cytosolic and endocytic roles in addition to mediating core membrane-fusion mechanisms required in cells. When fibroblasts from Down syndrome patients were subjected to serum starvation to induce autophagy, LC3 and P62 levels increased, autophagic lysosomes accumulated, and autophagic flux was impaired. Meanwhile, the expression of SNARE proteins STX17 and VAMP8 decreased, and after overexpression of STX17 or VAMP8, autophagic degradation in fibroblasts was restored, and P62 expression was downregulated [38]. Dijie Zheng et al. [39] found that prodigiosin could block late autophagy in cholangiocarcinoma cells through the SNAREs complex pathway. STX17 and SNAP29 increased in this experiment after rat hepatocytes were exposed to various concentrations of Cd, most likely due to the increased expression of membrane fusion proteins brought on by the buildup of autophagosomes. Meanwhile, the co-localization of LC3 with both STX17 and SNAP29 was reduced, indicating that Cd could inhibit the transfer of STX17 to autophagosomes, which in turn affected the formation of STX17–SNAP29 binary complexes. After Cd exposure, the expression of VAMP8, a SNARE protein localized on the lysosome, gradually decreased with increasing Cd concentration, and the co-localization of VAMP8 with LAMP2, a lysosomal membrane protein, was significantly reduced. It indicates that Cd inhibits the expression of VAMP8 while reducing the amount of VAMP8 on lysosomes. According to Jean S et al. [40], cell starvation enhanced the interaction between the MTMR13 DENN structural domain and RAB21, promoting the activity of RAB21 and its interaction with VAMP8 and upregulating the translocation of VAMP8 to endosomes. This results in increased VAMP8 endolysosomal trafficking and thereby promotes autophagosomal fusion with lysosomes. In the present study, we hypothesized that Cd inhibited the translocation of VAMP8 to endosomes, thereby reducing the VAMP8 endolysosomal trafficking. Co-treatment of Tau with Cd increased the co-localization of LC3 with both STX17 and SNAP29, as well as the co-localization of VAMP8 with LAMP2. It indicates that Tau can promote the transport of STX17 to the autophagosome and, thus, the formation of the STX17–SNAP29 binary complex, as well as the transport of VAMP8 to the lysosome.

## 4. Materials and Methods

### 4.1. Reagents

Cadmium chloride, taurine, anti-LAMP2, anti-LC3B, and anti-p62/SQSTM1 were purchased from Sigma-Aldrich (202,908, St. Louis, MO, USA). DMEM and FBS were obtained from Gibco (Grand Island, NY, USA). Alexa Fluor 488-labeled goat anti-rabbit IgG, Cy3-labeled goat anti-rat IgG, Lyso-Tracker Red (LTR), and Hanks’ Balanced Salt Solution were obtained from Beyotime (Shanghai, China); Anti-β-actin, anti-Atg7, anti-Beclin-1, anti-CTSB, and anti-rabbit IgG were obtained from Cell Signaling Technology Inc (Danvers, MA, USA). Anti-LAMP2 was obtained from Santa Cruz Biotechnology (Santa Cruz, CA, USA). DQ-BSA-Green was purchased from Invitrogen (Carlsbad, CA, USA). Anti-STX17, anti-SNAP29, and anti-VAMP8 were obtained from Abcam (UK). All of the other materials were of analytical grade.

### 4.2. Cell Culture

The Institute of Biochemistry and Cell Biology’s Cell Bank (Shanghai, China) provided the BRL3A cells. Cells were grown in DMEM supplemented with 10% FBS, 100 U/mL penicillin, and 100 mg/mL streptomycin and then incubated at 37 °C in a 5% CO_2_ atmosphere.

### 4.3. Analysis of Cell Proliferation

The real-time cell analysis system (Roche Applied Science, Basel, Switzerland) and 5-Ethynyl-2′-deoxyuridine (EdU) Cell Proliferation Kit with Alexa Fluor 488 were used to determine the proliferation of BRL3A cells. BRL3A cells were seeded at a density of 10,000 cells/well in 100 μL medium aliquots in quadruplicate; cells were analyzed every 15 min. Cells were treated according to the experimental design when the average cell index reached approximately 2.5. The results were normalized at the time of treatment. For EdU staining, BRL3A cells were seeded in 24-well plates and cultured to approximately 80% confluence, followed by treatment with Tau and/or 5 μM Cd for 12 h. After treatment with Pue and Cd, the cells were incubated in EdU working solution for 2 h, followed by the manufacturer’s treatment guidelines of 4% paraformaldehyde, 0.3% Triton X-100, Click Additive Solution, and Hoechst 33342, in that order. Finally, images were captured under a Leica DMIRB inverted microscope (DMI3000B, Leica, Wetzlar, Germany). Cells positive for EdU were counted as proliferative cells.

### 4.4. Analysis of Cell Morphology

BRL3A cells were seeded in 24-well plates and cultured to approximately 80% confluence, followed by treatment with 80 mM Tau and/or 5 μM Cd for 6 h. Then, cells were photographed with the Leica DMIRB inverted microscope. The doses of Cd and Tau were determined based on previous studies [30,41,42,43]. While referring to the previous studies, we also screened the concentration of Tau, and finally 80 mM Tau was used to treat the cells in this experiment.

### 4.5. Transmission Electron Microscopy

BRL3A cells were seeded in 6 cm dishes, grown to a confluence of about 80%, and then exposed to 80 mM Tau and/or 5 μM Cd for 12 h. The cells were fixed with 2.5% glutaraldehyde and osmium tetroxide, dehydrated in graded ethanol, immersed in a Spurr resin, and stained sequentially with uranyl acetate and lead citrate. Finally, autophagosomes were observed by a transmission electron microscope (CM 100, Philips, Holland).

### 4.6. Western Blotting Analysis

After treatment, the cells were lysed and extracted by ultrasonic RIPA, buffered, and washed with PBS. Total cell protein concentration was determined using a BCA protein assay kit from Beyotime in China. The same quantity of total protein was separated using SDS-PAGE and transferred to the PVDF membrane. After sealing the membrane for 2 h at room temperature with 5% skimmed milk, incubate the membrane overnight at 4 °C with the primary antibody. After 12 h, the diluted secondary antibody was incubated at room temperature for 2 h. Finally, the protein band was observed using the enhanced chemiluminescence test kit, and its gray value was examined using Image Lab software (National Institutes of Health, Bethesda, MD, USA).

### 4.7. LTR Staining

The cells were given a 12 h course of treatment with 80 mM Tau and/or 5 μM Cd, followed by 30 min of incubation with LTR working solution at 37 °C, during which time fluorescence images were recorded using a Leica DMIRB inverted microscope (DMI3000B, Leica, Wetzlar, Germany).

### 4.8. Analysis of Lysosomal Degradation Capacity

BRL3A cells were seeded in a confocal disk and cultured to about 80% confluence. Cells were pretreated with 10 μg/mL DQ-BSA Red for 2 h and then treated with 80 mM Tau and/or 5 μM Cd for 12 h. Finally, after staining with Hoechst 33342, the fluorescence image was quickly captured under a fluorescence microscope (TCS SP8 STED, Leica).

### 4.9. Infection and Analysis of StubRFP-SensGFP-LC3 Lentivirus

We obtained the stubRFP-sensGFP-LC3 lentivirus from GeneChem Corporation (Shanghai, China). After being cultivated to a confluence of about 20% in 24-well plates, BRL3A cells were co-incubated with stubRFP-sensGFPLC3 lentivirus for 48 h. Then, the positive cells were chosen using 4 g/mL puromycin (Solarbio, Beijing, China) to create stable cell lines. The GFP-RFP-LC3-marked cells were seeded on glass coverslips in 24-well plates. The complete medium was replaced with serum-free media at 80% confluence, and the cells were then treated in accordance with the guidelines of the study. Then, the fluorescence images of LC3 puncta were viewed using a fluorescence microscope (TCS SP8 STED, Leica). The yellow LC3 puncta indicate autophagosomes, and the red LC3 puncta indicates autolysosomes.

### 4.10. Immunofluorescence Staining

In 24-well plates, BRL3A cells (3 × 10^4^ cells per well) were planted for 12 h on the glass coverslips. Following several procedures, double staining was carried out as follows. First, after fixation with 4% paraformaldehyde, the cells were incubated separately with 0.5% Triton X-100 and 5% bovine serum albumin. Next, the cells were incubated with anti-LC3B antibodies diluted 1:200 in 5% BSA, together with anti-LAMP2 antibodies diluted 1:100 in 5% BSA overnight at 4 °C. Then, the cells were incubated with secondary antibodies labeled with Alexa Fluor 488 and Cy3. Finally, a fluorescence microscope was used to view the cell fluorescence images after staining with 4′,6-diamidino-2-phenylindole.

### 4.11. Statistical Analysis

Results were presented as the mean ± standard deviation (SD). Statistical data comparisons among groups were performed using a non-parametric, one-way analysis of variance (ANOVA), with *p* < 0.05 considered statistically significant.

## 5. Conclusions

This study provides the first cellular information about how Tau can facilitate the transfer of the SNARE proteins STX17 and SNAP29 to autophagosomes and the translocation of VAMP8 to lysosomes. This facilitates the fusion of autophagosomes with lysosomes, relieves blockage of autophagic flux, and alleviates Cd-induced damage to BRL3A cells. Tau may also boost lysosomal degradation and promote lysosomal acidity simultaneously.

## Data Availability

The datasets used or analyzed during the current study are available from the corresponding author on reasonable request.
